# Strategies in surface engineering for the regulation of microclimates in skin-medical product interactions

**DOI:** 10.1016/j.heliyon.2024.e25395

**Published:** 2024-02-01

**Authors:** H. Reuvekamp, E.E.G. Hekman, E. van der Heide, D.T.A. Matthews

**Affiliations:** a Laboratory for Surface Technology and Tribology, Department of Mechanics of Solids, Surfaces and Systems (MS3), Faculty of Engineering Technology, University of Twente, Postbox 217, 7500 AE Enschede, the Netherlands; b Biomedical Device Design and Production Lab, Department of Biomechanical Engineering (BE), Faculty of Engineering Technology, University of Twente, Postbox 217, 7500 AE Enschede, the Netherlands

**Keywords:** Surface engineering, Microclimate regulation, Personal healthcare device, Contact interface, Skin-product interaction

## Abstract

There is a growing number of personal healthcare devices that are in prolonged contact with the skin. The functionality of these products is linked to the interface formed by the contact between the medical apparatus and the skin. The interface can be characterised by its topology, compliance, and moisture and thermal regulating capabilities. Many devices are, however, described to have suboptimal and occlusive contacts, resulting in physiological unfavourable microclimates at the interface. The resulting poor management of moisture and temperature can impact the functionality and utility of the device and, in severe cases, lead to physical harm to the user. Being able to control the microclimate is therefore expected to limit medical-device related injuries and prevent associated skin complications. Surface engineering can modify and potentially enhance the regulation of the microclimate factors surrounding the interface between a product's surface and the skin. This review provides an overview of potential engineering solutions considering the needs for, and influences on, regulation of temperature and moisture by considering the skin-medical device interface as a system. These findings serve as a platform for the anticipated progress in the role of surface engineering for skin-device microclimate regulation.

## Graphical abstract

**Figure:**
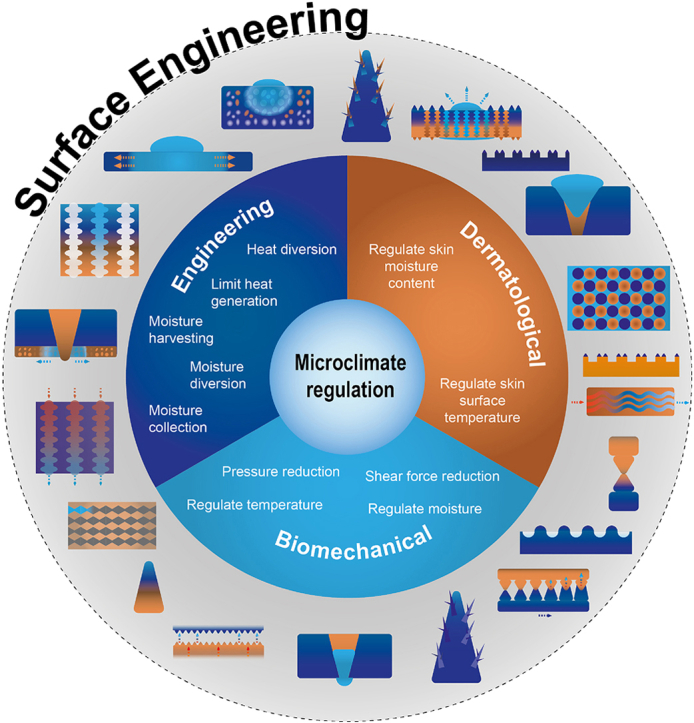

## 1 Introduction

At various places on the body, minimally invasive medical products are worn to promote the user's health [1]. These products include visual and audio aids, electrodes, devices for intestinal stoma's, activity trackers, orthoses and protheses [[2], [3], [4], [5]] and perform different roles: from passive support by restoring a bodily function [3,6], to more advanced monitoring of physiological parameters [2,3,[5], [6], [7]]. Based on the definition by Leonhardt [3] and Van Houten [8], this work defines a personal healthcare device as “*a minimally invasive, wearable medical product* supporting *the user throughout the day in any given situation or environment*”.

For any personal healthcare product to be able to achieve its function, a contact between the body and the device is needed. This connection is defined as the skin-product interface, which forms part of the skin-device system. From a mechanistic approach, the system can be characterised by four main factors: topology, compliance, and moisture and thermal regulating capabilities. These factors are interrelated, and the system is transient. The resulting combination of the parameters at each side of the interface alters the area and the type of contact between both sides. A partially conformal contact between the two sides creates an occluded environment at and around the interface. Consequently, this leads to conditions conducive to the generation and accumulation of heat, moisture and/or (bodily) waste. The region combining these thermal and moisture elements is in literature also referred to as a microclimate region [1]. Prolonged and occlusive contact with an adverse microclimate cannot only lead to skin irritation [9,10], contact dermatitis [11] and medical-device relates injuries (MDRI's) [12], but also any physiological measurements performed by a device can be impacted [2,13]. All of these complications adversely affects the health of- and the comfort experienced by the user.

To limit MDRI's and sustain a healthy skin barrier, the microclimate elements must be regulated. It is advised that optimal skin conditions are maintained, and the origins are treated [14]. Current solutions are primarily focused on mitigating MDRI's and rehabilitating the skin condition, rather than preventing them by sustaining an optimal microclimate region at the skin-medical device interface. This can be attributed to an incomplete understanding of the impact of occlusion, specifically in the context of a microclimate, on (moisture-associated) skin damage [14]. Solutions focusing on engineering a surface, by means of texturing, coating and/or material development, contributing to a physiological favourable microclimate, are suggested, but rather limited in medical device-related practice. However, due to the influence of the device side and the surface and material properties characterising it, surface engineering for microclimate control is a valuable direction to explore. It is hypothesised that this will enable the interface to be engineered in such a way that it ensures an optimal microclimate. To develop the features at the device-side in such a way that the device can have a regulatory effect on the microclimate, it is paramount to understand all thermal and moisture driven effects happening at the skin-device interface, and their influence on the (bio-)mechanical properties of the skin-device system.

Therefore, the main aim of this study is to review the current knowledge concerning microclimate regulation at the interface between the skin and various personal healthcare devices. To investigate the interface-related phenomena systematically, a well-accepted and structured method known as the systems approach [15] will be employed. This approach involves dividing the system into multiple interconnected elements, each of which is influenced by operational conditions.

The review will start with characterising the system elements based on three distinct perspectives: the dermatological perspective which focuses on the skin side; the engineering perspective, which pertains to the device side; and the biomechanical perspective which concerns the microclimate. Subsequently, the systems elements will be related to delineate the influences and resultant effects that are present in this system. This serves as the foundation for establishing requirements, necessary functionalities, and targeted values, all of which are significant in considering surface engineering as a potential microclimate management strategy. Furthermore, this review will explore surface engineering strategies that have been applied in other application domains, which can be used within the context of regulating the microclimate at the skin-personal healthcare device interface. In considering the existing challenges, also opportunities for prospective advancements are discussed, which may support the use of surface engineering strategies for microclimate regulation.

## 2 The elements of the skin-device system

**Fig. 1:**
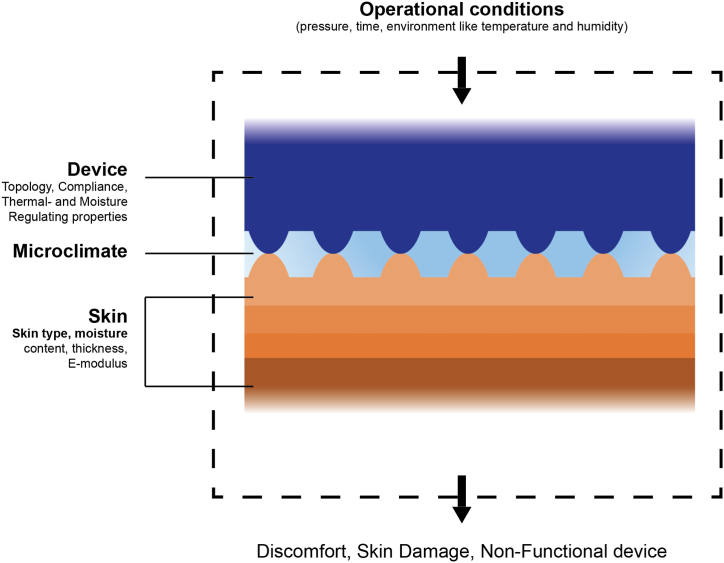
Schematic representation of the skin-device system, consisting of three main components: the device-side, the skin-side and the intermediate layer comprising the microclimate. Each component can be characterised by multiple properties. Depending on the operational conditions like the surrounding ambiance, the exerted pressure, and contact time, various consequences such as discomfort, skin damage and non-functional devices can occur.

Interactions observed at the skin-device interface are system dependant. To illustrate, the topology and compliance of the skin, being one side of the interface, can vary due to contact with moisture [[16], [17], [18]]. In turn, by varying these parameters at the device side via engineering strategies, the resulting functionality can be influenced as well. Following the systems approach, two sides of the interface can be defined: the skin side and the device side, as depicted in Fig. 1**.**

### 2.1 The dermatological perspective: the skin-side

As the largest and most externally exposed organ in the human body, the skin primarily acts as a barrier to protect inner tissues from injury, drying out and penetration of foreign substances [[19], [20], [21], [22], [23]]. In addition, it senses the environment, and it transports, for example, water contents in- and outwards by sweating, and regulates the body temperature [19,20,24]. These functions are achieved by the multi-layered skin consisting of the epidermis, the dermis and the hypodermis [21]. The most superficial layer of the epidermis, the stratum corneum (SC), provides the skin's rigidity and is described as the main performer of the skin's barrier function [[25], [26], [27]]. In general, each layer holds specialised contents creating functions to withstand a variety of influences like mechanical stresses, the presence of variable (excessive) moisture, temperature or chemical substances [28].

The skin and its mechanical and protective performance can be characterised by multiple properties such as the roughness, thickness, elasticity, hydration, sweating rate, sebum secretion and transepidermal water loss (TEWL) [20]. TEWL is the ease with which the skin allows internal water passing through the upper layers to the surrounding, dryer atmosphere via passive diffusion and evaporation [26,29,30]. These characteristics of the skin are interpersonal and evolve throughout a person's lifetime [20,31]. To illustrate, with increasing age, there is a decrease in the overall hydration level due to a lower amount of natural moisturising factors present in the skin [32,33]. The roughness, characterised by the dimensions of the skin features, and its anisotropy increases due to aging [34]. Other intrinsic factors influencing the skin characteristics are gender, ethnicity, skin type, anatomical site, body mass index, lifestyle and the presence of various diseases [20,32,33,35]. Extrinsic factors such as the ambient temperature and relative humidity (RH), the season of the year or the circadian rhythm also impact the skin function [[36], [37], [38], [39]]. Moreover, all properties of the skin are interdependent and operate as one system [20]. Hence, alterations of one skin parameter can lead to changes of other interrelated aspects.

### 2.2 The engineering perspective: the device-side

In this paper, the counter side of the system is formed by the personal healthcare product being in contact with the skin. As limited examples, in the upper region, facemasks, visual and audio aids are the most significant products [2,3]. Moving on towards the torso, one finds clothing, devices for intestinal stoma's, medical tapes and band aids and various types of electrodes [[2], [3], [4], [5]]. At the upper extremities, casts, gloves, and activity trackers can be present [2,3,5]. Urinary, menstrual, and sexual aids are used on or around the genitals. Products in contact with the lower extremities are for example protheses and orthoses and urine collection bags being part of a catheter system [40,41]. At the feet, several devices supporting and monitoring health conditions such as diabetic feet can be found [[42], [43], [44]]. Typical materials used for these personal healthcare devices are polymers and textiles [2,40,45]. Common polymeric materials include stiff (methacrylic) plastics [11,46,47], and more flexible thermoplastic elastomers such as silicone [40,47,48], (thermoplastic)polyurethane (T) (PU) [45,49], polydimethyl-siloxane (PDMS) [2,49,50]or polyvinyl chloride (PVC) [47,51]. Additionally, ethylene-vinyl acetate (EVA), polypropylene (PP), and polyethylene (PE), are used, with the latter sometimes applied as a foam. Additionally, non-woven fabrics composed of materials like nylon and cellulose are found in medical products due to the mechanical flexibility they provide [45]. Another observable trend as regards materials is the use of (stronger) adhesives or overtapes to increase the life span of a device [[52], [53], [54]].

### 2.3 The biomechanical perspective: the microclimate

The region at and around the skin-device interface in which moisture and heat accumulate is often named a microclimate. The concept of a microclimate was first introduced in literature in 1953, where several studies investigated the microclimate underneath clothing [55,56]. This area between a textile and the skin is still considered a microclimate in literature [[57], [58], [59], [60]]. Other contexts for which the presence of a microclimate is described include dermatitis [61,62], diabetic feet ulcer [63] and pressure ulcers [[63], [64], [65]].

The microclimate is often referred to as a local region differing from the surrounding ambiance with respect to temperature, airflow, and humidity [1,64,66,67]. However, the description of this region and the elements characterising it are approached differently throughout literature. For example, The National Pressure Ulcer Advisory Panel characterises the microclimate as “*the local tissue temperature and moisture (RH) level at the body/*support *surface interface*” [68]. Gefen [69,70] describes the microclimate in the context of pressure ulcer development, with the skin surface temperature (T_ss_), RH, moisture and air movement being important components. Temperature is frequently cited as the primary contributor to skin damage resulting from the formation of a microclimate in various definitions since it can induce variations in the moisture component through processes such as perspiration [71,72]. Additionally, temperature has been shown to be a good predictor of pressure ulcers [73]. In today's research, both temperature and humidity in all forms are considered as the main elements contributing to skin damage resulting from skin-device interactions [42,63,[74], [75], [76], [77], [78], [79]]. The moisture component can be formed by multiple aspects such as TEWL, humidity in the air and other skin moisture generating mechanisms such as sweat, wound exudate or leakage of bodily fluids [[80], [81], [82]]. The presence, and hence contribution of airflow within the microclimate is a point of discussion. The reasons for this are twofold. Firstly, air movement can be used as part of a microclimate management strategy [1,83]. Secondly, the microclimate can be defined either a closed or open region. For the system defined in this paper, the microclimate is considered as the *“local region between the device and the skin comprising the combined effects of temperature and humidity which are deviating from the surrounding ambiance”*. This is in accordance with the early observations of Roaf [84] who was one of the first mentioning the microclimate and its great influence on the aforementioned system's components and resulting MDRI's for example. In Roaf's study, the need to maintain a favourable microclimate is emphasised. This can be achieved, predominantly, by regulating the temperature and moisture components within the skin-device system.

## 3 Combining the perspectives: the resulting effects and targeted values

**Fig. 2:**
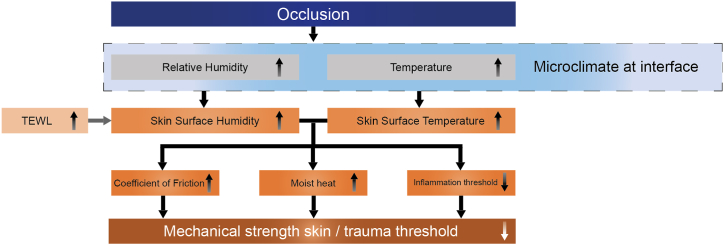
The effects resulting from skin occlusion, adapted from Ref. [1] with permission from Elsevier (License no. 5641380567704). Arrows inside the text blocks indicate an increase (upwards arrow) or decrease (downwards arrow) of the respective parameters.

The regulation needed is based on the interactions between both sides of the system. This is determined by the individual properties described, as well as by the resulting contact between the skin and the product. The initiation of skin damage stems from the application of a device on the skin, leading to the formation of a microclimate that promotes moisture and heat accumulation. Skin occlusion, in and of itself, is not necessarily problematic, as the skin can adapt to its environment and recover its original state [71,85]. However, prolonged covering of the skin changes its mechanical properties and decreases its barrier function, rendering it more vulnerable to applied loads, ultimately leading to the development of MDRI's such as dermatitis and pressure ulcers [86,87]. Fig. 2, as adapted from Kottner et al. [1], provides a summary of these consequences and their associated effects.

### 3.1 Occlusion

Occlusion arises by covering the skin with impermeable, non-breathable materials often used in personal healthcare products such as plastics and textiles. Also, the use of tapes, moisturizers and gels makes the skin being enclosed by a microclimate [87]. The size of this resulting environment can be regarded as the difference between the nominal contact area and the real contact area. Within this area various conditions are described. A thermo-neutral microclimate refers to the range of ambient conditions in which the human body is capable of maintaining its core temperature by regulating dry heat loss [88]. These circumstances are considered to be ∼23 °C/∼50 % RH [89], as also specified in ISO554-1976 (E) [67,89]. These microclimate values can change, due to occlusion. Hot-humid and a hot-dry microclimates are mentioned to be ∼28–37 °C with a RH of ∼80 % and ∼42 °C with a RH of ∼20 %, respectively [[89], [90], [91]]. The microclimate's RH is dependent on several factors, including the insulation properties of the medical device, the physiological activity of the skin, and the RH of the surrounding environment [92]. The ideal RH at the skin-product interface is described to be between 40 and 65 % [93,94] and the skin surface temperature (T_ss_) should not change by more than a few degrees Celsius. These values should be therefore targeted when considering an optimised regulation of the microclimate.

### 3.2 Skin temperature change

The T_ss_ is subject to exogenous and endogenous changes resulting from numerous factors, such as internal body temperature, thermal properties of the tissues, blood flow, moisture content, and metabolism. These factors collectively contribute to the surface of the human body being a rich map of isotherms [95]. In ambient conditions typically ranging from 14 to 29 °C, a regular T_ss_ is usually found to be between 29 and 33 °C [93,[95], [96], [97], [98]]**.** The mean T_ss_ can be approximated by

(1) T_ss_ = 23.44 + 0.32*T_A_ [90]

Equation (1). Mean skin temperature as a function of ambient air temperature in Celsius [90]

with T_A_ being the ambient temperature in Celsius. The skin temperature calculated with this equation can be considered the regulation goal with regards to the thermal component of the microclimate.

Furthermore, the skin temperature responses are affected by thermal, mechanical and surface properties of both the skin and counter side made up by the device [99]. For example, during short term occlusion, within 20 min after covering, the first effect to be noticed is that the skin temperature rises to ∼37 °C [89,100,101]. This was observed in studies where covered contact resistors measured an increase of T_ss_ between 1.3 and 4.5 °C [44,89]. Also, the effect of clothing thermal resistance on the skin temperature as investigated by Liu et al. [102] who showed that all skin temperatures, and especially those of the feet, were higher with an increased resistance. Based on these outcomes, it can be assumed that covering the skin surface restricts heat loss [89], and results in an increase in T_ss_. Prolonged contact of a personal healthcare device with the skin not only impedes heat loss, it also exerts pressure or loading on the skin, thus restricting blood flow [85]. Additionally, there is often a thermal gradient between the skin-side and the device-side [103]. Both of these effects contribute to a change in T_ss_. Studies [99,104] examining changes in skin temperature during contact, within a force range of up to 2 N, have indicated that the thermal contact resistance varies with contact force, and has a significant effect on the temperature of the skin. This contact resistance in the interface is responsible for the difference in temperature between the skin and the device side, hence the heat conduction during this contact [99,105]. To further estimate the (initial) surface temperatures of both the skin and device side of the system, the contact resistance allows evaluation of the heat flux in steady-state condition between the skin and a rigid surface at a known temperature [105]. With no fluid in the interface region, the thermal contact resistance between two surfaces can be described using surface parameters as the roughness and asperity slope, and mechanical bulk material properties such as the thermal conductivity, the hardness and contact pressure exerted [99]. Examining these relationships shows that the surface related aspects greatly influence the temperature change. The bulk material properties of the device side remain constant for example when being in contact with moisture, whereas the microhardness and roughness aspects of the skin side of the system are changed. Long term occlusion, defined as being after 24 h of covering, is for example described to change the skin of the volar arm: The secondary lines, partially making up ‘polygonal’ network structure, disappeared, the polygonal geometry is lost and the roughness of the skin decreases [1]. In the end, this results in a changing contact resistance, and ultimately a skin temperature response.

### 3.3 Skin humidity change

Likewise, both short- and long-term occlusions enhance the moisture content in and around the skin-device interface. A lack of evaporation is considered the primary reason. An occlusion experiment conducted to observe the effects of limiting evaporation revealed that high humidity levels were attained within an hour, particularly in the presence of a water layer on the skin [106]. Insulated and non-permeable materials made the RH, for instance, around the foot rise by 13.2 % [44]. Observations showed that after 20 min of sitting and 30 min of walking more sweat accumulated under these materials, compared to the permeable materials [44]. Other studies discussed in Ref. [93] showed that the RH, as measured under the seated buttocks, varied between the physiological preferred value of 40 %, up to 100 %.

It is well documented that the moisture content of the SC depends on the RH and temperature of the environment [107,108]. In healthy skin, the SC typically has a water content of 10 – 20 % [101]. The moisture sources leading to an increase in skin hydration and RH are mainly TEWL and sweating [109]. Sweat glands are activated at ambient temperatures above 30 °C [110]. By impeding evaporation, the sweating process also fails as a cooling mechanism. Research has shown that TEWL is strongly associated with skin temperature and RH, with the latter appearing to be the more significant factor. This suggests that the amount of moisture available for TEWL is actively regulated in response to changes in environmental conditions [36]. Hence, whenever T_ss_ increases, the upper critical value is dependent on the lack of evaporation, rather than the metabolic rate [111].

### 3.4 Mechanical strength and trauma threshold

The change in both moisture content and temperature because of prolonged occlusion disturbs the skin in numerous ways. For instance, skin temperatures above 35 °C are described to affect the mechanical stiffness and strength of the SC [67,112]. Likewise, the moisture causes a swelling of the corneocytes in the SC [16] and an increase in SC thickness [16,17,113] which disrupts the lipid structure, resulting in less withstanding of stress in this layer. Overhydration also alters mechanical properties [16,108,114] like the elastic modulus, a parameter reflective of the mechanical behaviour of the most important body protector, the SC [17]. For example, there is a factor 10 reduction in the elastic modulus of human SC when the RH was increased from 30 to 95 % [107]. In tensile tests with animal SC, the Young's modulus has been shown to decrease from 887 dyne/cm^2^ to 1.2 dyne/cm^2^ when the RH increased from 26 to 100 % at 25 °C [115]. In nanoindentation experiments, the Young's modulus decreased from ∼100 MPa to ∼10 MPa if the conditions changed from dry to wet [116].

Another side-effect of (long-term) occlusion is the enhanced penetration ability of potentially irritant substances into the skin leading to an increased risk for irritation and contact dermatitis in severe cases [87,117]. Typically, occlusions result in increased temperature and humidity in a local area of the skin. This changes the skin structure, which leads to a reduced permeability, a higher coefficient of friction (CoF), and a higher susceptibility to forces and irritating substances [118]. One of the most described consequences of this lower trauma is (shear and normal) loading induced tissue damage such as pressure ulcers.

### 3.5 Impact of microclimate elements on resulting effects of prolonged suboptimal contact

The extent to which the described microclimate elements, heat, and moisture, play a role in the resulting consequences of prolonged contact with an unfavourable microclimate is limitedly covered in literature. Numerous studies were performed to understand the effect of the two most prominent factors, temperature, and humidity, on the CoF. For example, Klaassen et al. [92] developed a friction map for skin-textile interaction which shows the dependence of friction, RH and temperature. Both RH and temperature increase the CoF. An explanation for the strong link between the three components is hypothesised to be the elevated water uptake at higher skin temperatures, leading to plasticisation of the SC and an increased skin compliance, and eventually a higher friction [92]. Hendriks and Franklin [91] showed that the friction was twice as high in a humid (80–90 % RH) environment. In the study of Dinc et al. [119] the CoF between fingertips and plexiglass increased by ∼20–30 % when the RH was increased from 35 to 90 %. This implies that the combined effect of heat and moisture has a considerable influence on the resulting damage, in combination with occlusion and applied forces. This stipulates the importance of maintaining a physiological optimal microclimate [92]. To do so, the ideal values described, such as the ideal RH and T_ss_, in the preceding sections should be targeted by two main functionalities: moisture and temperature regulation.

## 4 Microclimate regulation

In general, the management of the microclimate elements and the resulting MDRI's poses challenges for both dermatology and engineering practices. The skin and its health are contingent on several factors and all skin properties operate as one interpersonal system. Ultimately, this makes it challenging to define and devise an optimal intervention targeting the skin component of the skin-product interface. While the microclimate can affect the mechanical characteristics of the device, this impact is relatively minor compared to that on the skin side. In fact, it has been shown that the device side can greatly influence the microclimate components and the resulting skin side of the skin-device system. To avoid impeding the skin's natural regulatory functions, the device must be capable of averting the resulting effects of (prolonged) occlusion, and thus the effects of moisture and temperature accumulation at the skin-device interface [14,81,83,120,121].

### 4.1 Dermatological and biomechanical perspective: current management strategies

**Fig. 3:**
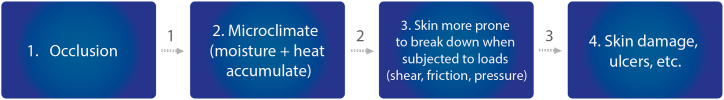
Sequence of cause and effects for occlusion (dark grey). The arrows (light grey) indicate the points at which a prevention strategy can be applied.

Fig. 3 provides an overview of the sequence of events resulting from the formation of an occlusion. Common interventions to prevent skin damage resulting from an occlusion are pressure relieving devices as support mattresses, and low-air-loss surfaces, which are particularly used to address heat and moisture build up [67]. These devices are favoured due to their lower labour and cost intensity compared to manual body repositioning [67,122,123]. Most of these solutions are focused on preventing or reducing effects such as pressure ulcers (indicated by arrow no. 3c in Fig. 3).

Promoting accelerated healing (block no. 4 in Fig. 3) of skin and wounds via wound debridement and reduction of the bacterial load is another strategy often applied [124]. This is achieved by using various impregnated, infused or hydrocolloid dressings and other strategies, such as uptake-variant components as absorbent pads and the use of lotions [124,125]. Hydrogels and dressings are the gold standard in wound management [125,126], as they create a suitable wound healing environment [126,127]. Their three-dimensional functional network possesses excellent and tuneable mechanical, thermal and biological properties, including stiffness and thermal conductivity, which correspond to that of native skin [126,128]. However, hydrogels are occlusive, preventing water vapour exchange between the wound and its surroundings [124]. This contradicts the advice for preventing physiological unfavourable microclimates [128]. Additionally, Orlov & Gefen [125] showed that certain dressings had an inferior wound exudate handling due to failed transport in direction perpendicular to the skin and no equal spreading within the absorbent material. Therefore, the use of dressings is only effective if underlying causes and risk factors such as shear forces, friction, and pressure are limited and if the fluid is managed effectively.

These findings suggest that most of the current interventions involve a combination of strategies and are not necessarily of full preventive nature. This stipulates the need to develop, firstly and foremost, an interference aimed at an earlier stage in the scheme depicted in Fig. 3. Another reason for finding alternative, technology-implemented strategies is the relative high costs associated with current management strategies [129]. In the pursuit of optimising intervention strategies and creating regulation functionalities, it is important to consider an additional facet, namely the realisation that adaptation of the bulk material is complex. Thereby, the resulting interface functionality is greatly linked to surface characteristics. By engineering the surface parameters, it is possible to add explicit functionalities to specific parts. Hence, surface engineering is hypothesised as a viable measure for regulating both moisture and temperature in skin-device contacts.

### 4.2 Engineering perspective: surface engineering as a potential design solution

In recent research, as summarised in multiple review papers [[130], [131], [132], [133], [134], [135], [136], [137]], applying surface engineering strategies without changing the shape or materials of the devices has shown the ability to create desired interface functionalities such as improved wettability, (anti)bacterial adhesion properties, friction and wear reduction, and optimising optical properties by structural coloration and anti-reflectivity. The strategies to achieve these functionalities have been described in various applications, or as stand-alone, unapplied concepts. Solutions focusing on engineering a product's surface contributing to a favourable microclimate are suggested [14], but rather scarce in medical device-related practice. However, surface engineering techniques are already employed for various other medical functionalities, such as imparting antibacterial properties [132,133,[137], [138], [139], [140]], improving the bio- and hemocompatibility [141,142], promoting cell culturing [132,[142], [143], [144]], enhancing the tribological performance [145], and facilitating tissue adhesion [146]. In other application domains such as automotive, tribology, aerospace and energy its effectivity for the creation of regulating microclimate elements has been proven [131,134]. For example, engineering surfaces by means of texturing is described to contribute to improved lubrication [[147], [148], [149]], an optimised CoF [[148], [149], [150], [151], [152]], minimised friction-induced structural degradation [147,151,153], controlled adhesion, or the regulation of the surface wettability [133,154].

To design and implement surface engineering for regulating the microclimate state for skin-personal healthcare device interfaces, general desirable functionalities can be formulated based on the aforementioned causes of the experienced MDRI's and physiological, unfavourable microclimate conditions. In general, there are two main requirements that need to be achieved. Firstly, the amount of moisture within the microclimate region must be managed [13,14,83,93,120,121,[155], [156], [157]]. The desired value within the microclimate should be kept between 40 and 65 % RH [93,94] and the skin's moisture content should be around 10–20 % [101] or 30–55 in corneometer units [20]. Secondly, temperature accumulation must be diminished to keep the T_ss_ within a physical favourable and comfortable range of 16–33 °C [83,88,90,[95], [96], [97], [98],^101^,^158^,^159^].

#### 4.2.1 Moisture regulation

Moisture management is the controlled movement of water vapour and liquids from a substrate to the atmosphere over and through a countersurface [160]. Regulation has already been employed in applications such as fog collectors [[161], [162], [163], [164], [165], [166]], sensing wearables [13,155,167,168], support surfaces [67,169], personal moisture management [162,170], wound dressings [80,120,[171], [172], [173]], and fabrics [83,162,[174], [175], [176], [177], [178], [179], [180]]. According to the research in these domains, to regulate the moisture content in the microclimate region, three sub-functionalities need to be performed consecutively: moisture harvesting (MH), moisture diversion (MD) and moisture collection (MC) [179].

**Fig. 4:**
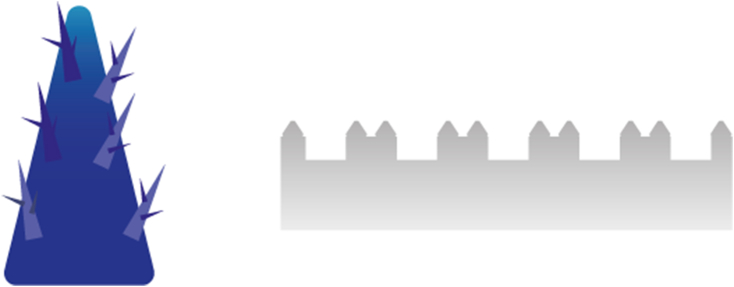
Hierarchical structures for Moisture Harvesting; Micro-trees (left) and a more general hierarchical design consisting of grooves with spikes on top (right).

**Fig. 5:**
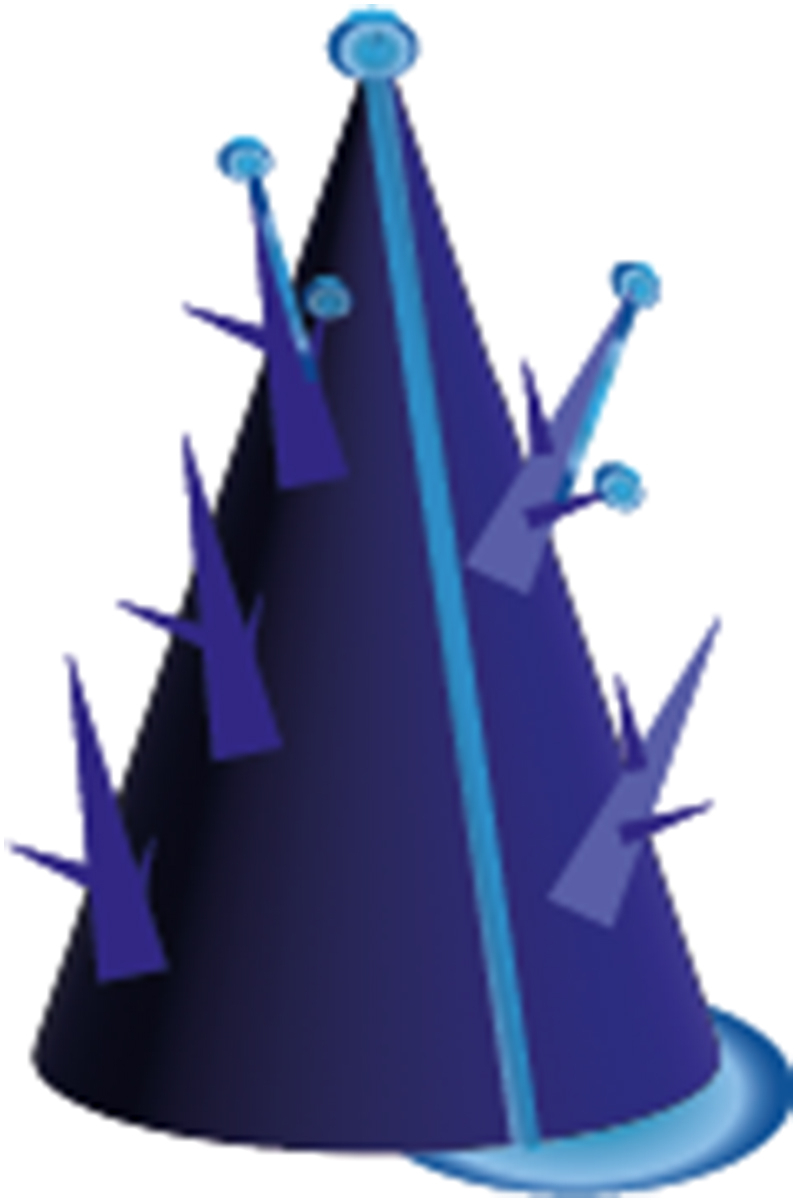
The physical phenomena underlying the moisture harvesting capability in a hierarchical structure.

**Fig. 6:**
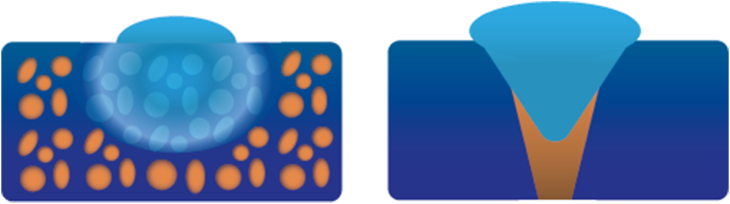
Pressure difference strategies for Moisture Harvesting. From left to right: Pores in porous material & A conical channel. The orange and blue colour depict different wettability's to create an optional wettability.

To effectively control moisture levels, it is essential to attract both gaseous and liquid states through MH. A collective approach to draw both types of humidity to a surface, is the use of a hierarchical structures [149,171,181,182]. Recent examples of such hierarchical structures include “micro-trees” [181], and more straightforward vertically-aligned spikes [182], strategically positioned on top of a substrate which can also be modified with a designed topology. Illustrations are presented in Fig. 4. The use of multiscale topographies has also demonstrated moisture harvesting abilities [2]. The underlying physical phenomena, as depicted in Fig. 5 driving MH in these two hierarchical structures revolve around the curvature gradient following from the tip shape facilitating the growth of microscale water droplets onto the surface. This, in turn, promotes continuous condensation of new droplets on the cones [181]. The resulting Laplace pressure difference moves the coalesced water droplets toward the ‘stem’ of the cone [182], thereby creating additional sites for subsequent droplet condensation [162]. Also, capillary pressure can be created, as shown in sweat collecting wearable devices, through conical nano or micropores present in porous materials like textiles [165] or foam. This effect is sometimes induced by combining capillary pressure build-up with- or by a stand-alone wettability difference, which spontaneously wicks sweat into hydrophilic microchannels [13]. This is also called an inlet-suction effect [149]. Fig. 6 depicts these pressure differences employed for MH.

**Fig. 7:**
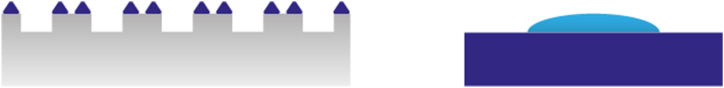
From left to right: A biphilic surface with hydrophilic (dark blue) nano bumps on a (super)hydrophobic (grey) surface with micro scale grooves & a single hydrophilic substrate with a droplet on top (light blue).

Using a patterned substrate, which for example, consists of sub millimetric grooves [183,184], is another solution to achieve MH. Additionally, a wettability difference can be employed for MH [162]. The use of a biphilic patterned substrate, when fog comes into contact with the outer layer of hydrophobic material, triggers tiny water droplets to spontaneously move towards the hydrophilic area. There, they merge and can be subsequently gathered [162]. Biphilicity can be created through chemical surface modifications, by constructing alternating hydrophilic and hydrophobic areas on a surface [164,185,186], or via a hierarchical structural design with length scales ranging from nanoscale to a couple of millimetres [184]. A example of a biphillic surface is found in Ref. [187], where hydrophilic nano bumps were placed on a superhydrophobic microscale patterned substrate, resulting in enhanced water collection flux compared to single surfaces due to balanced nucleation and transport enhancement. Nevertheless, single hydrophilic substrates are also used for MH [13,80,173]. These two strategies are illustrated in Fig. 7.

**Fig. 8:**
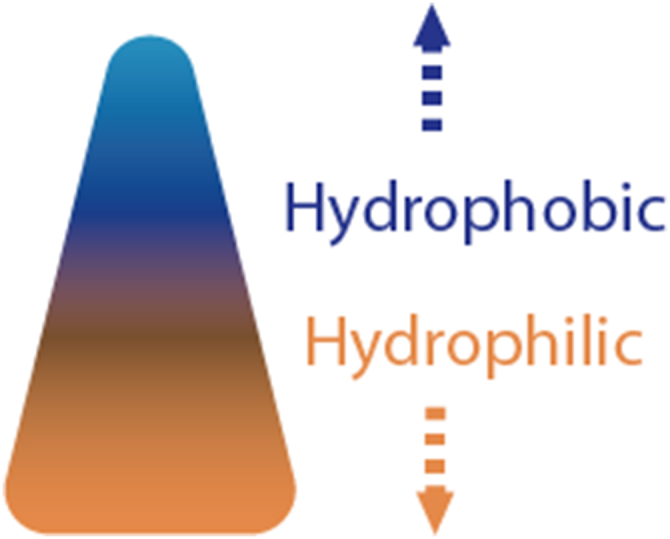
A wettability and structural gradient combined for Moisture Harvesting purposes.

Currently, a trend is observed in which wettability and structural gradients are combined [[162], [163], [164],^168^,^184^,^188^]. For instance, super hydrophilic, triangular patterns are produced on a superhydrophobic substrate [164] or tips characterised by a vertical wettability gradient along the conical shape [166] (Fig. 8) are employed for MH.

**Fig. 9:**
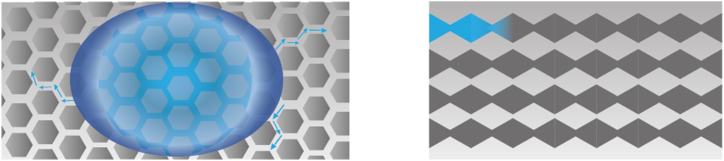
Top view of a patterned surface resulting in a set of connected microchannels (left) and a top view of an asymmetrical channel structure (right).

Subsequently, the harvested moisture must be transported to a more favourable location. The concept of MD can be perceived as passive, directional liquid transport. Similarly to MH, passive movement is frequently accomplished through the utilisation pressure differences, like a wettability- or a structure gradient [180]. A wettability gradient for this functionality is, for instance, created by a chemical modulation of the surface, resulting in a biphilic pattern [189], as depicted in Fig. 8. A structural design can also be seen as a topographic design is the application of microgrooves and microcavities [190]. Structure gradients to achieve a pressure difference are accomplished by having a patterned substrate. For example, triangular shaped patterns [164], or a set of microchannels [182,190,191] create a Laplace pressure gradient, allowing for passive moisture transport. Moreover, the use of an asymmetric channel structure can create capillary action directing the liquid towards, for instance, sensors on a wearable sweat collector [13], or microfluidic devices [192]. This is shown in Fig. 9.

Porous materials like foams, nonwoven acquisition material, or fibers applied are used for the pressure gradient resulting from the asymmetric structure of the connected pores and their size variations [171], the latter being depicted in the left illustration in Fig. 6. These materials facilitate passive moisture transport as well [162,170,178].

**Fig. 10:**
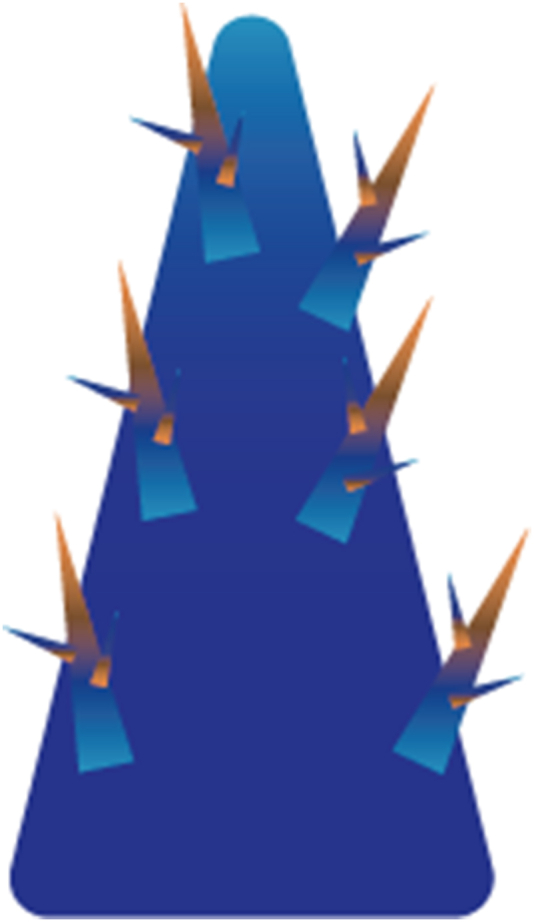
A conical shape covered with smaller cones. The colour gradient on these smaller spines depict a wettability pattern.

**Fig. 11:**
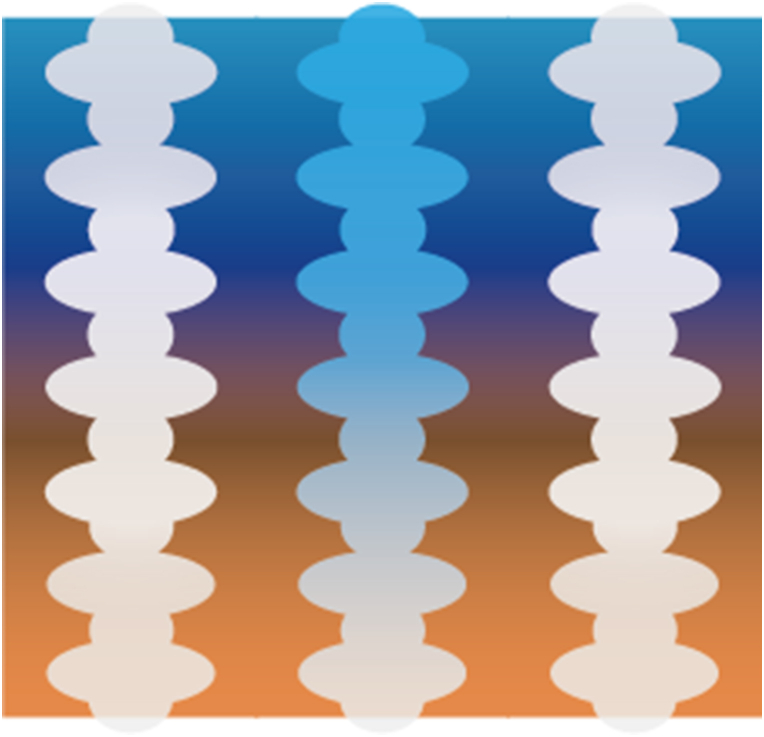
Meta fabric for directional liquid transport. The porous structure (grey) transport the liquid. The fabric has a hydrophilic and hydrophobic side as shown by the orange-blue gradient.

**Fig. 12:**
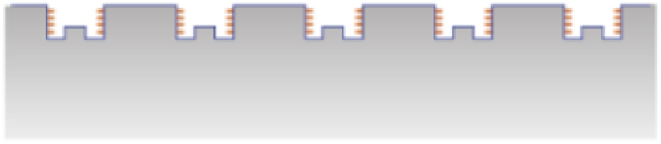
Multilevel channels. The bottom of the main channel is covered with hydrophilic grooves and ridges (dark blue). The ridges are covered with hydrophobic structures (orange).

A variation in pressure can also be achieved by combining a wettability variation with structural modifications. Firstly, this can entail the integration of both a wettability and a structural gradient, as already shown in Fig. 8. An example is the bioinspired structured surface for fog collection which features conical spines, comparable to a cactus, which are covered with a biphilic pattern, based on the back of desert beetle [166]. The harvested droplets move toward the hydrophilic spots due the force induced by the wettability gradient. The gravity and curvature gradient further aid this transport [166]. An illustration in Fig. 10 depicts this surface design. Secondly, a biphilic, porous structure emergers as another option. For instance, in the meta fabric designed for epidermal electrodes, the porosity gradient resulting from the fiber network and the wettability asymmetry, create directional liquid transport [155], see Fig. 11. In another study, the fabric undergoes plasma treatment to create localised wettability gradients [178]. The alternating wettability regions, combined with the through-thickness or pore size variations, facilitates continuous and passive liquid transport [162,165,170,178]. A good example of a hierarchical, biphilic structure are fiber networks, consisting of fibers which exhibit either hydrophilic or hydrophobic properties [80,180]. Hierarchy is achieved through the introduction of micro-sized bumps onto the fibers, thereby significantly enhancing their wettability to an extreme degree [180]. Alternatively, the combination of multiple singular networks on top of each other [80] can be regarded as hierarchy. The arrangement of the biphilic fibers results in capillary action [80,180]. In epidermal patches, multilevel channels with varying hydrophobic and hydrophilic surfaces are also observed [168]. The bottom of channels are covered with hydrophilic grooves and ridges, whereas the ridges also have rough, granular structures on top of them, rendering them hydrophobic [168], as illustrated in Fig. 12. A hierarchical structure can also independently induce a Laplace difference. This has been shown in the wicking behaviour of open-microchannels from which the side-walls were covered with fin-like structures [192]. These current employed strategies are in line with the advice given by the panel members consulted in the article of Gray et al. [14] and Gefen et al. [193]. These recommendations include controlling or diverting of the moisture source [14,193]. Another suggestion involves the prevention of skin contact with extensive and irritant substances to prevent (severe) MDRI's. The engineered surface or material should be able to directionally steer it into more preferable directions, or to absorb and gradually release liquids [147].

**Fig. 13:**
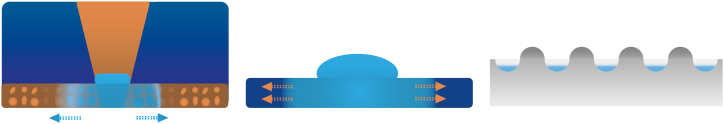
From left to right: Absorbing and storing moisture via porous structures, absorbing and storing via hydrophilic dressing, a patterned substrate holding moisture.

Consequently, following capturing and transporting moisture, it must be collected in a way that is does not affect the microclimate or either side of the interface. Ultimately, it is critical to avoid excessive drying at the skin-device interface, as it may lead to reduced tissue integrity resulting from decreased levels of lipids, water content, elastic modulus, and weakened junctional integrity between skin layers [194]. Collecting consists of absorbing, storing, but also releasing the moisture. For the two former sub functionalities porous structures and/or hydrophilic materials are predominantly used. In both the context of fog harvesting and moisture management in fabrics, hydrophilic foams, dressings and fibers, create a force attracting the moisture [120,127,155,163,173]. This is depicted in the two most left schematics in Fig. 13. These porous structures and hydrophilic dressings, as well as patterned substrates (the most right illustration in Fig. 13) provide micrometer spaces in which moisture can be held. For example, a keratin hydrogel is combined with nanofibers for absorbing wound exudate [127]. Micro grooves can also retain lubricant [147], or in other words liquid, at the surface. Due to the unidirectional nature of this process, moisture release occurs on the outer surface of the system through either passively or by actively removing the liquid via evaporation. In the case of fabrics, this process is governed by liquid transportation to the outer side, where it spreads across the surface and where it can evaporate due to thermal differences and airflow [120,156].

**Fig. 14:**
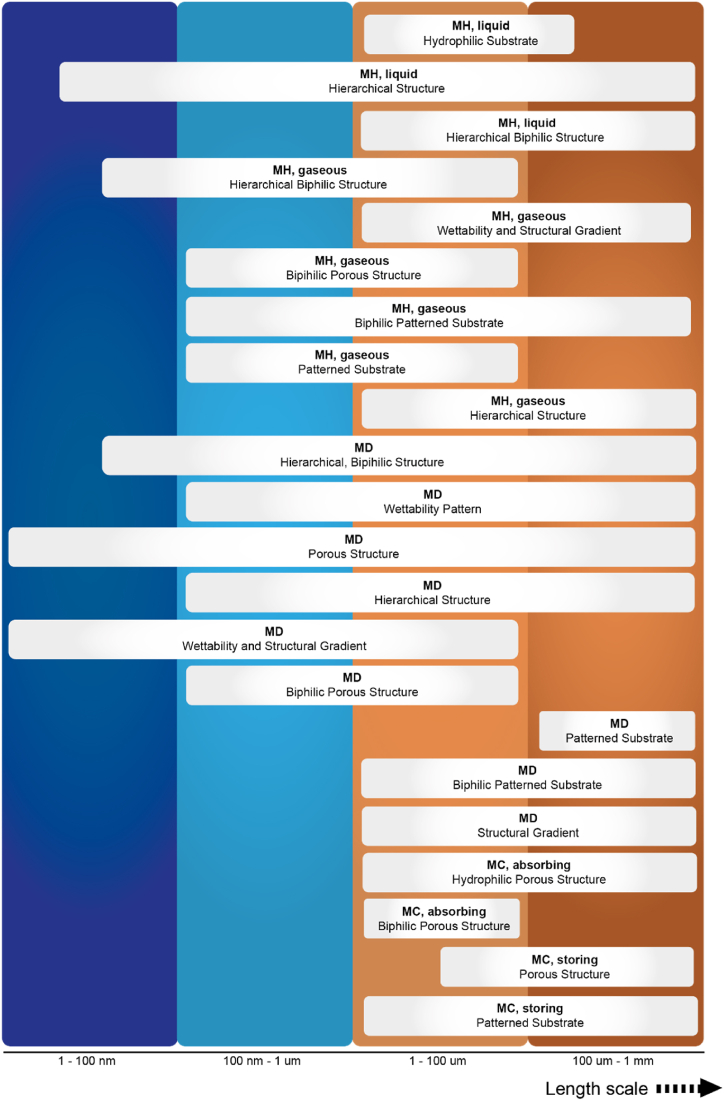
Overview of illustrated moisture regulation strategies, Moisture Harvesting (MH), Moisture Diversion (MD) and Moisture Collection (MC), and their associated length scales.

In summary, there are currently three main strategies employed to achieve sub-functionalities for regulating the moisture component in the microclimate region. The first strategy involves establishing a wettability difference, while a second strategy is based on the generation of a Laplace gradient [180], both of which can be considered pressure gradients. A third approach integrates these two strategies, resulting in a synergetic effect [162]. These strategies are achieved through topology design that involves altering the size, shape and spatial distribution of surface features, and/or by varying the material properties [162,163,188,190]. The use of hierarchical structures is accompanied by careful consideration of dimension compatibility. Fig. 14 illustrates the specific strategies presented and discussed above, with their respective length scales.

#### 4.2.2 Thermal regulation

In general, controlling the thermal component of the microclimate region involves two critical aspects: limiting the generation and the transportation of excessive heat accumulating at the skin-device interface. Products, such as micro-electronical products [[195], [196], [197], [198], [199]], heat exchanger equipment [200,201], protheses [202,203] and textiles [169,179,180], are currently employing strategies aimed at mitigating an unfavourable temperature at a product interface.

**Fig. 15:**
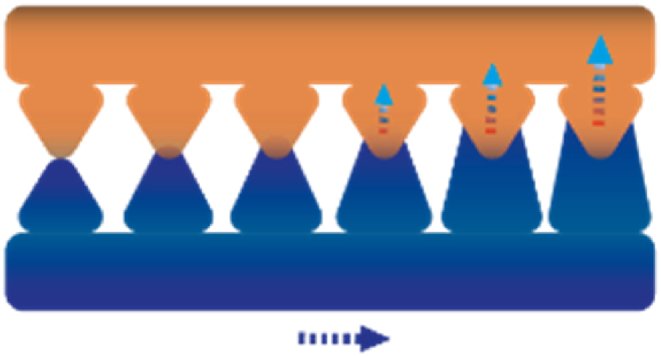
Enhanced heat transfer by enlarging the contact area between two system sides.

**Fig. 16:**
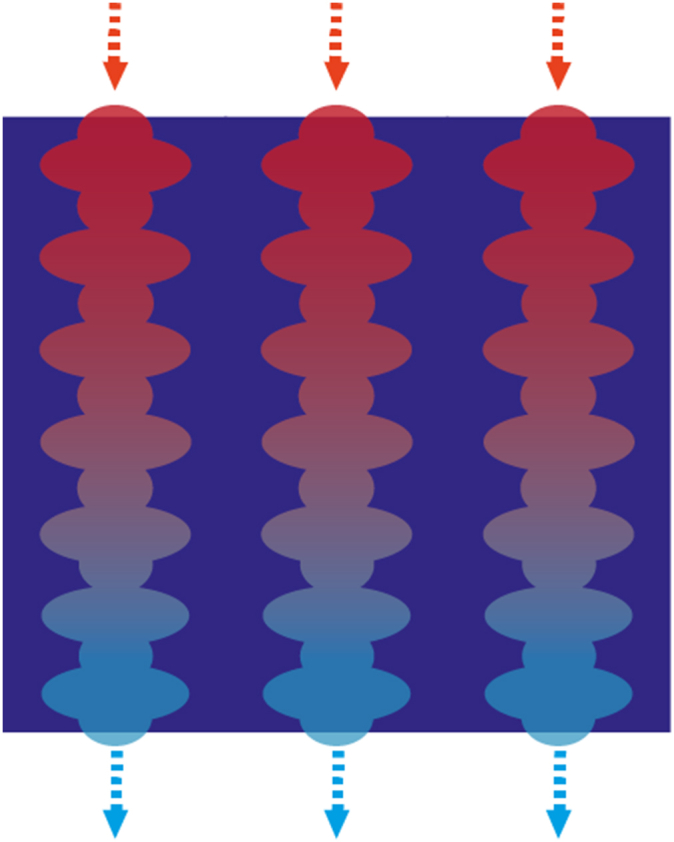
Open Porous Structure to limit the generation of excess heat.

In the context of skin-device interfaces, thermal regulation is done by limiting the heat being generated (LoHG) or by heat diversion (HD). One way to mitigate heat generation involves the application of a pattern onto a substrate. This approach is particular used in the context of manufacturing tools, where optimised friction is considered as a main heat generation mechanism. Texturing a surface can retain and provide lubricants for example to reduce friction [147], constraining heat production. However, the effectiveness of this technique is contingent upon the scale of the texture; research has demonstrated the superiority of micro/nano-scale textures over sub-millimeter sizes [147]. The surface contact area is also enlarged, which reduces the contact resistance and improves heat transfer across the interface [198,202]. This is depicted in Fig. 15. Alternatively, the generation of excess heat can be limited by adopting an open, porous structure, as illustrated in Fig. 16. Utilising spaced fabrics, for instance, enhances air permeability and lowers thermal resistance [169].

**Fig. 17:**
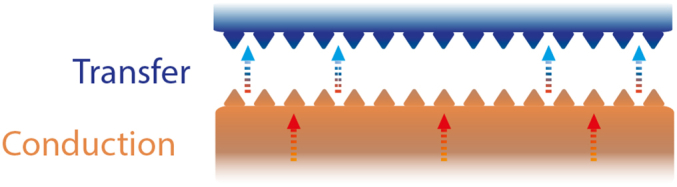
Effective temperature diversion through heat transfer and conduction.

**Fig. 18:**
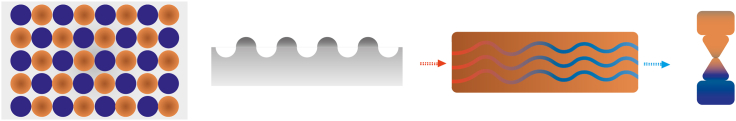
From left to right: passive heat diversion through a biphilic pattern, passive heat transfer through a patterned substrate with dimples, passive heat transfer through passages and passive heat transfer by matching the thermal conductivity to the desired properties of the counterside where blue depicts the device side and orange the skin-side of the skin-device system.

HD is also approached through more effective heat transfer and conduction (Fig. 17). Current research has concentrated on heat transfer mechanisms using vapour chambers [195,199], as well as vapour condensation facilitated by wettability gradient resulting from a bipihilic pattern [44]. Patterned substrates also play a role in enhancing flow through geometric means such as the utilisation of dimples [200] or wedge shaped tracks [44]. Furthermore, increased HD can be achieved by optimising the thermal conductivity of materials [79]. For instance, Williams et al. [202], altered the thermal conductivity of liner materials to lower the socket temperature. Gefen [128] proposes a specific approach for optimising the heat transfer. It underscores that the thermal conductivity, as well as the surface feature sizes, should align with values of the human skin to enhance the heat transfer between both surfaces. This alignment is important, because significant variations in thermal conductance between contacting materials results in a temperature gradient. Consequently, a considerably lower thermal conductance hinders heat transfer, turning it into an insulating barrier [128]. These strategies have been summarised in the illustrations of Fig. 18.

**Fig. 19:**
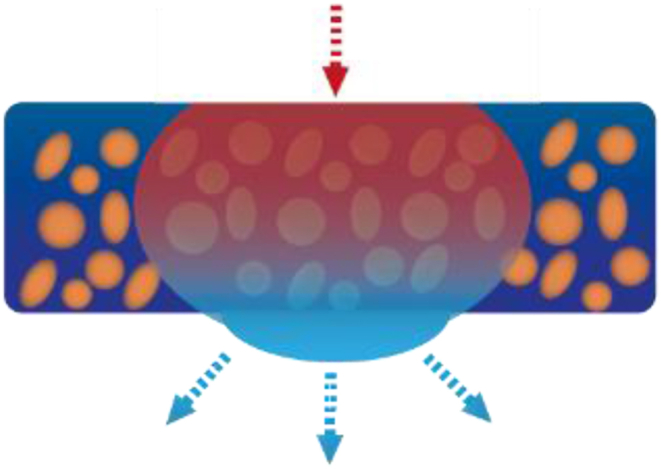
Open structure for air permeability. Orange depicts a different wettability compared to the dark blue colour.

**Fig. 20:**
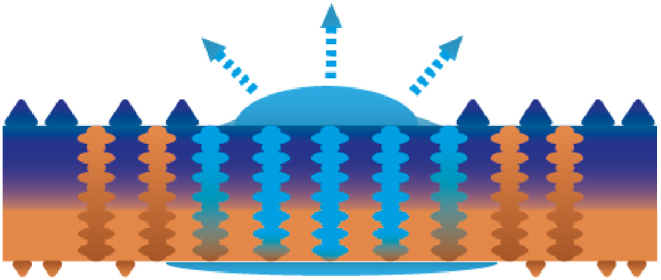
An illustration of the evaporation principle by a porous structure. The blue and orange colour display the varying wettability's.

**Fig. 21:**
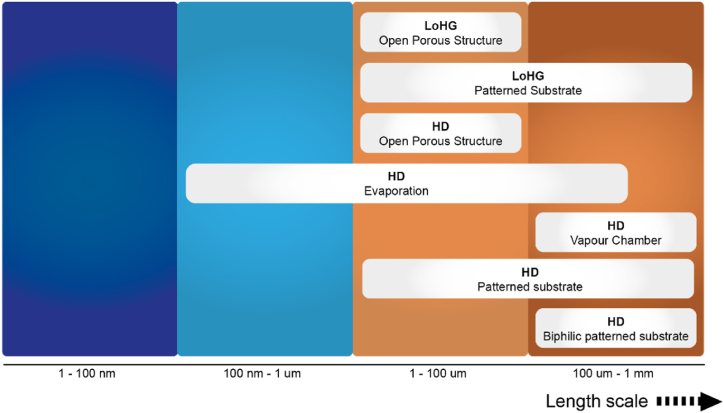
Overview of illustrated thermal regulation strategies, Limitation of Heat Generation (LoHG) and Heat Diversion (HD), and their associated length scales.

Frequently, the use of a biphilic, porous structure (Fig. 19) accomplishes both thermal management sub functionalities (LoHG and HD) simultaneously. In the research of Dong et al. [155], a porosity gradient is achieved by constructing a layered structure of fibers with different diameters. Also, these fibers are composed of different materials, rendering the electrode fabric with wettability gradients. A similar approach is observed for a Janus membrane recently developed by Wang et al. [180], where various textiles are employed on opposing sides to establish distinct hydrophilic and hydrophobic regions. To further promote the wettability, one side of the membrane is covered with globular micrometer scale bumps, while the other side has nanoscale bumps. The physical phenomena governing this thermal management is the directional liquid transport, comparable to sweat pumping [180]. As illustrated in Fig. 20, the alteration in fiber diameter creates asymmetrical channels inducing capillary action. Once the liquid has reached the outer surface, the fabric supports spreading across the surface where it can evaporate [156]. The exothermic nature of evaporation results in heat loss [156], thus leading to a dry and cool microclimate [180]. All these strategies for regulating the thermal component have been summarised in Fig. 21, including the associated length scales, following from all the sources in Table 1. The thermal conductivity has been left out since this is related to bulk material properties rather than a designated length scale.

**Table 1:** Surface functionalities and potential strategies for microclimate regulation.

Main functionality		Target value	Sub functionality		Design solutions from other applications
Microclimate regulation	Regulate moisture content in microclimate region [13,80,81,83,120,121,155,157,162,173,[178], [179], [180],^204^]	• Relative humidity surrounding interface between 40 and 65% [66,93] • Moisture content in the skin 10–20% [101] or 35–55 corneometer units [20]	Moisture harvesting [13,80,162,168,171,173]	Liquid [13,80,162,168,171,173]	• Hydrophilic substrate [13,80,173] • Hierarchical structure [149,171] • Hierarchical, biphilic structure [164,168]
				Gas	• Hierarchical, biphilic structure [162,163,184,188] • Wettability and structural gradient [166] • Biphilic, porous structure [165] • Biphilic patterned substrate [162,164,[184], [185], [186]] • Patterned substrate [183,184] • Hierarchical structure [181,182]
			Moisture diversion [13,14,80,81,155,156,[167], [168], [169], [170], [171],[178], [179], [180],^204^]		• Hierarchical, biphilic structure [80,163,168,180,188] • Wettability pattern [13,190] • Porous structure [13,81,156,169,180,199,204] • Hierarchical structure [13,149,190,192] • Wettability and structural gradient [13,162,166,167,180] • Biphilic, porous structure [155,162,165,170,171,178,179,204] • Patterned substrate [147,164,182,190,191] • Biphilic patterned substrate [189,190] • Structural gradient [190]
			Moisture collection [120,127,156,171,173,193]	Absorbing [120,127,171,173,193]	• Hydrophilic, porous structure [163] • Biphilic, porous structure [155] • Hydrophilic dressing [120,127,173]
				Storing [120,127,193]	• Porous structure [163,171] • Patterned substrate [147] • Hydrophilic dressing [120,127]
				Releasing [120,156,173,193]	• Evaporation [120,156,173]
	Regulate temperature content in microclimate region [79,83,121,128,[155], [156], [157],^169^,^179^,^180^,^204^]	• Skin surface temperature 16–33 °C [88,93,95,96,101,158,159,205]	Limitation of heat generation [155,169,180,204]		• Open, porous structure [155,169,180,204] • Patterned substrate [147]
			Heat diversion [79,128,155,156,169,179,180,202]		• Open, porous structure [79,155,169,202] • Evaporation [156,179,180] • Vapor chamber [195,199] • Optimal thermal conductivity [79,128,202] • Patterned substrate [200] • Biphilic patterned substrate [191]

Table 1 presents a summary of prospective surface engineering strategies with the potential to function as design solutions for microclimate regulation. The solutions are classified according to the fundamental surface designs employed for achieving the intended functionalities. The first three columns present the main- and sub functionalities necessary to attain targeted values for a physiologically, favourable microclimate at the skin-device interface. These contents stem from sources having a dermatological and biomechanical focus and/or are written in the context of skin-device interfaces.

The table reveals that while considerable attention has been devoted to main functionalities related to microclimate regulation at the skin-device interface, relatively limited progress has been made in addressing sub functionalities for microclimate regulation in this context. In contrast, other application domains offer a plethora of potential solutions. Within these solutions, which are presented in the last column of Table 1 and it is important to distinguish between a patterned substrate, a biphilic patterned substrate, a hierarchical structure and a structural gradient. These terms, while suggesting analogous surface configurations, exhibit subtle distinctions.

**Fig. 22:**
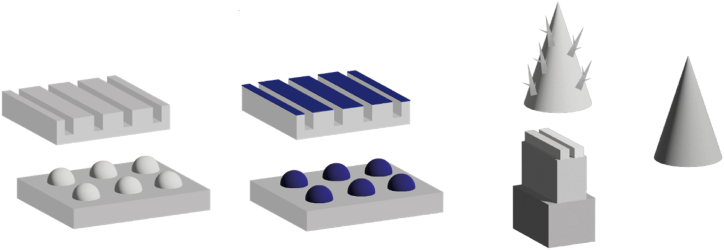
From left to right: Patterned substrates, Biphilic Patterned substrates, Hierarchical patterns and a structure with a structural gradient. The blue colour indicates a different wettability compared to the grey colour.

As illustrated in Fig. 22, a patterned substrate is characterised by geometrical features, such as grooves [183] or bumps, arranged in a specific pattern on top of a flat surface. In contrast, a biphilic pattern deviates from this configuration by replacing the geometrical features with spots with a differentiating surface energy compared to that of the bulk material of the substrate. For instance, Hou et al. [164] implemented hydrophilic triangular patterns on a hydrophobic substrate, while Gukeh, Damoulakis & Megaridis [191] selectively removed hydrophobic material to create wedge-shaped patterns onto a superhydrophilic substrate. Conversely, a hierarchical structure comprises of multiple geometrical features with varying length scales integrated in vertical arrangement. Noteworthy examples include the micro tree-like structures of Shi et al. [181] and the nanograssed micropyramids from Chen et al. [188]. A structural gradient or curvature gradient [162] also shows variation in vertical direction, but consists of singular one-length scale features such as the cones used on top of a channel structure by Kim et al. [182] or needles utilised by Bai et al. [163].

### 4.3 Microclimate regulation characterisation techniques

The described design solutions employed in other application domains have shown effective outcomes. Due to the various and differentiating nature of these commonly studied surface engineering strategies, these are hard to compare to determine the most optimal strategy and parameters. To do so, and to enable reproducibility and make outcomes applicable to all contexts, and in particular for and within the microclimate region or on the device side of the skin-device system, quantifiable and reproducible characterisation must take place.

Characterisation of the microclimate condition in practice and laboratory settings differs across studies. There exists a considerable variation in the aspects measured and utilised for the determining the current state of the microclimate. The state can be measured directly by determining temperature and/or RH [67,81,106,206] or indirectly by determining physiological values of the skin such as the hydration of the skin [73,81,106], T_ss_ [70,73], and TEWL [66,81,106]. Next to direct and indirect measurements, the considered factors used to characterise the state of the microclimate can also be subdivided in different categories based on the three perspectives: microclimate (engineering) measures like the temperature and RH in the microclimate region, (bio)mechanical measures including pressure forces and the elastic modulus of both interface sides, and biophysical (dermatological) measures such as the TEWL [63], pH, skin moisture content, and T_ss_. These skin values, such as TEWL, pH, the thickness and the hydration of the SC are aspects which can represent the skin barrier function [14,20,25,66,81,106], which in turn is influenced by the microclimate. For instance, the pH is influenced by the skin hydration, including sweat secretion and activity, and environmental conditions [25]. A healthy skin has a mildly acidic to neutral pH value varying between 4 and 6 [14,25]. Due to the passive diffusion and evaporation guiding TEWL, this value largely depends on the skin temperature and environmental humidity [207,208]. The higher the skin temperature or the lower the ambient humidity, the higher the TEWL value becomes [38,209,210]. For this reason TEWL, in particular the rate of evaporation of water from the skin surface, is considered as an indirect parameter to assess the state of the microclimate [66]. Similarly, T_ss_ is considered an indirect microclimate property since it is altered by prolonged contact with the microclimate. This is for example shown by two studies [70,71] in which the microclimate is characterised based on assessing elevated T_ss_ underneath dressings and its entropy by employing infrared thermography.

Despite multiple measurements being conducted to characterise the specific aspects mentioned, accurate comparison of the findings remains a challenge. One of the reasons is the fact that most of the current assessments are focused on properties at the skin-side of the interface which are personal and greatly influenced by intrinsic and extrinsic properties. For instance, TEWL an indirect measure depicting the skin diffusion, while the TEWL instruments measure the evaporation flux [66]. Additionally, TEWL is unstable since it is easily distorted by breathing and air flow. Another challenging factor is the short acclimatisation time of human skin. In studies typically a time between 10 and 30 min is being used [29,58,91,209,[211], [212], [213]]. These factors create difficulty in obtaining consistent, reproducible and normalised results. Sometimes the skin properties such as the TEWL are directly assessed after removal of the pad [81]. In contrast, measuring microclimate properties such as the temperature and RH are at the same time measured directly with 30-min intervals by inserting a sensor in-between both system sides [81]. Another way to overcome heterogeneity of the skin is performed by Call et al. [67] who used a body phantom for assessing both humidity and temperature at the device side of a skin-device interface during a laboratory study. A limitation of this approach is the neglect of certain skin-related factors such as perspiration, other moisture effects, and vasodilation. Moreover, it should be noted that a set-up can greatly affect the resulting values, and consequently the definition of the microclimate that results from these measurements. For example, in some studies the temperature sensors are covered by tapes to secure them to the body or by placing the wafer-thin sensor between the skin and the device covering it [81], whereas in other studies these sensors are subjected to the free air. Another example is the emitting or storing of heat when using Infrared Thermography devices [70]. Also, for evaluating the same parameter, for instance TEWL, several types of systems, including open, closed, and ventilated rooms, can be used [66]. All of this can have a considerable influence on the defined state of the microclimate.

Likewise, there is a considerable variation in the dimensions described for measuring the state of the microclimate. To determine TEWL values, the size of a thimble, with an average height of 20–25 mm, is often used for measurement chambers [66]. Nilsson [214] considered that the boundary layer of air adjacent to the skin is ∼10 mm, which is consistent with the study of Kottner and Imhof [66]. For direct microclimate measurements such as temperature and RH in the microclimate region, dimensions are not specified in literature. The thickness of the layer making up the size of the microclimate region should be considered but is currently unspecified. This highlights the need to keep the dimensions constant when performing microclimate measurements. The variation in chamber construction illustrates the importance of the choices made regarding the set-up like reducing disturbances and sensor location, due to possible interference and significant influences on the outcomes, and definition of the microclimate following from this.

These observations reveal that current methods employed for experimental and research purposes are challenging owing to the physical constraints imposed by the set-up and measurement techniques. Although the set-ups and measurements provide a certain degree of quantification as regards the state of the microclimate region, the research process and applied methods show the need for further refinement in the (standardised) characterisation of the microclimate region [76] at the skin-device interface. Quantification of both temperature and humidity is essential in accurately assessing the microclimate and should be incorporated, either directly or indirectly, as key aspects.

## 5 Future outlook

Evaluation of the currently used design solutions from other applications shows the potential of applying surface engineering interventions for microclimate regulation at the skin-medical product system. This can be owned, among others, to the applied length scales and observed materials, as well as the functionalities proven in previous studies. However, the use of surface engineering strategies on personal healthcare devices for microclimate regulation is not straightforward.

The surface functionality that is required for the desired applications is highly correlated with the design process and its involved choices [152,215,216]. Given the complex requirements for achieving an optimal microclimate at the skin-device interface, a functional surface capable of performing multiple functionalities is necessary. Although outcomes have been shown in studies, future research needs to assess all discussed surface parameters and evaluate their performance with regard to the desired regulation functionalities and microclimate regulation context. It also remains to be determined whether a surface engineered for one specific function can also enhance the performance of other functionalities, thus raising the question of possible synergistic effects for microclimate regulation at the skin-device interface. This has also been demonstrated in Table 1, where design and underlying physical phenomena are existing for multiple (sub)functionalities. To illustrate, transport of perspiration is a known strategy for regulating the moisture amount on the skin, but also for controlling the body temperature [217,218]. However, implementing this functionality on the device side in a passive way, has yet minimally been demonstrated, and often only in textile-based materials such as Janus membranes [179,180,219,220]. In addition, continued research on hierarchical, biomimetic, responsive or intelligent surfaces, as well as (meta)materials [59,130,133,162,171,180,[221], [222], [223], [224], [225], [226], [227]] is expected to support the development of these multifunctional surfaces for this skin-device interface microclimate regulation.

To support the engineering process for utilising surface engineering to regulate the microclimate at the skin-device interface, general engineering guidelines should be formulated. The design solutions presented in Table 1 are proven for a specific application and material, which mostly differs from the context being the focus of this paper. It is suggested that future work should concentrate on creating design maps for various aspects, such as materials and contexts, to design surface regulation mechanisms towards an optimised extent [149,152,215,228,229]. Such a standardised procedure or prediction tool for surfaces with relation to the functionality is currently considered to be extremely limited [147,152]. However, some examples are present, such as the function and surface parameter maps constructed by Whitehouse [230]. These depict the role product surfaces can have, such as influencing the thermal conductivity and dry friction, and suggesting appropriate texture parameters to select for the performance of these functions.

To match the thermal and mechanical regulating functionalities with the application, device evaluations become increasingly important as well [128]. To make informed decisions for regulating the microclimate, an overview of the effects of surface engineering should be made which is based on both the development and optimisation through mathematical methods and experiments. Obtained numerical results should be compared with experimental data to validate and further optimise the overall design process of surface parameters [149].

With regard to testing, one overall standardised laboratory scale testing method assessing the microclimate conditions and the regulation ability of various surfaces being part of the skin-device system, is currently lacking. Both temperature and moisture levels are mentioned as useful aspects to quantify the microclimate and the effects of personal healthcare devices on the body [67]. First steps for a general method to test the surface performance for microclimate regulation can be made by defining uniform terminology and aspects to evaluate, which allows for comparison. This can include combining a set of already existing, standardised tests being used over the years which, for example, determine the effect of a support surface [67,75,76,78,93,94,231,232], dressings [233] or heat loss analysis of fabrics [156,169] including temperature and humidity dissipation. Additionally, multiple length scales should be taken into account, since micro- and nanostructures may exhibit different functionalities compared to bulk materials [234]. Therefore, a set-up which can measure at various length scales is considered a necessary addition to assess all microclimate interactions, as no such setup has been reported, to the best of the authors' knowledge.

## 6 Conclusion

This review aimed to provide a concise overview of the factors concerning the interactions taking place when the skin is in prolonged contact with a personal healthcare device, as well as the resulting consequences. Dermatological, biomechanical, and engineering perspectives collectively indicate that both moisture and thermal regulating functions need to be achieved. This is crucial to maintain a microclimate within a physiological, favourable range, preventing MDRI's. The utilisation of surface engineering is discussed as a promising strategy for controlling these microclimate components at the skin-medical device interface. This is primarily attributed to the ability to introduce specific and targeted functionalities to the device-side of the interface, allowing for customisation across various products and materials.

Controlling an optimal moisture level in the microclimate should be done through subsequent moisture harvesting, moisture diversion and moisture collection. Potential surface engineering strategies within other contexts show that moisture harvesting can be performed by applying hierarchical structures and biphilicity resulting in wettability and pressure gradients. Moisture diversion uses both wettability and structural gradients for passive transport. To absorb, store and eventually release the captured moisture, hydrophilic properties and porous structures can be used. Regulating the temperature content in the microclimate is achieved through limiting heat generation and transporting the heat. By enhanced contact with the ambient environment and optimising, for example friction components, less heat is produced. To divert heat, both conduction and transferring mechanisms can be applied through chemical and physical surface modifications. To conclude, the microclimate can be regulated by applying nano- and micro length scale surface engineering strategies at the device side of the skin-personal healthcare device system. Future work should involve investigation of the optimal parameters and application of these surface engineering methods within the personal healthcare device domain for microclimate regulation.

## Author contributions

Hanneke Reuvekamp: Conceptualization, Investigation, Methodology, Visualization, Writing – original draft, Writing – review & editing. Emile van der Heide: Supervision, Writing – review & editing. Edsko E.G. Hekman: Conceptualization, Supervision, Writing – review & editing. David T.A. Matthews: Conceptualization, Supervision, Writing – review & editing

## Data Availability statement

No data was used for the research described in the article.

## Ethics statement

No funding was received for this research.

## Declaration of competing interest

The authors declare that they have no known competing financial interests or personal relationships that could have appeared to influence the work reported in this paper.
